# Supervised Machine Learning Models for Predicting Sepsis-Associated Liver Injury in Patients With Sepsis: Development and Validation Study Based on a Multicenter Cohort Study

**DOI:** 10.2196/66733

**Published:** 2025-05-26

**Authors:** Jingchao Lei, Jia Zhai, Yao Zhang, Jing Qi, Chuanzheng Sun

**Affiliations:** 1 Third Xiangya Hospital of Central South University Changsha China

**Keywords:** sepsis, sepsis-associated liver injury, machine learning, stacking ensemble model, clinical decision support

## Abstract

**Background:**

Sepsis-associated liver injury (SALI) is a severe complication of sepsis that contributes to increased mortality and morbidity. Early identification of SALI can improve patient outcomes; however, sepsis heterogeneity makes timely diagnosis challenging. Traditional diagnostic tools are often limited, and machine learning techniques offer promising solutions for predicting adverse outcomes in patients with sepsis.

**Objective:**

This study aims to develop an explainable machine learning model, incorporating stacking techniques, to predict the occurrence of liver injury in patients with sepsis and provide decision support for early intervention and personalized treatment strategies.

**Methods:**

This retrospective multicenter cohort study adhered to the TRIPOD+AI (Transparent Reporting of a Multivariable Prediction Model for Individual Prognosis or Diagnosis, Extended for Artificial Intelligence) guidelines. Data from 8834 patients with sepsis in the Medical Information Mart for Intensive Care IV (MIMIC-IV) database were used for training and internal validation, while data from 4236 patients in the eICU-Collaborative Research Database (eICU-CRD) database were used for external validation. SALI was defined as an international normalized ratio >1.5 and total bilirubin >2 mg/dL within 1 week of intensive care unit admission. Nine machine learning models—decision tree, random forest (RF), extreme gradient boosting (XGBoost), light gradient boosting machine (LightGBM), support vector machine, elastic net, logistic regression, multilayer perceptron, and k-nearest neighbors—were trained. A stacking ensemble model, using LightGBM, XGBoost, and RF as base learners and Lasso regression as the meta-model, was optimized via 10-fold cross-validation. Hyperparameters were tuned using grid search and Bayesian optimization. Model performance was evaluated using accuracy, balanced accuracy, Brier score, detection prevalence, F1-score, Jaccard index, κ coefficient, Matthews correlation coefficient, negative predictive value, positive predictive value, precision, recall, area under the receiver operating characteristic curve (ROC-AUC), precision-recall AUC, and decision curve analysis. Shapley additive explanations (SHAP) values were used to quantify feature importance.

**Results:**

In the training set, LightGBM, XGBoost, and RF demonstrated the best performance among all models, with ROC-AUCs of 0.9977, 0.9311, and 0.9847, respectively. These models exhibited minimal variance in cross-validation, with tightly clustered ROC-AUC and precision-recall area under the curve distributions. In the internal validation set, LightGBM (ROC-AUC 0.8401) and XGBoost (ROC-AUC 0.8403) outperformed all other models, while RF achieved an ROC-AUC of 0.8193. In the external validation set, LightGBM (ROC-AUC 0.7077), XGBoost (ROC-AUC 0.7169), and RF (ROC-AUC 0.7081) maintained strong performance, although with slight decreases in ROC-AUC compared with the training set. The stacking model achieved ROC-AUCs of 0.995, 0.838, and 0.721 in the training, internal validation, and external validation sets, respectively. Key predictors—total bilirubin, lactate, prothrombin time, and mechanical ventilation status—were consistently identified across models, with SHAP analysis highlighting their significant contributions to the model’s predictions.

**Conclusions:**

The stacking ensemble model developed in this study yields accurate and robust predictions of SALI in patients with sepsis, demonstrating potential clinical utility for early intervention and personalized treatment strategies.

## Introduction

Sepsis is a complex and multifaceted syndrome resulting from the body’s dysregulated response to infection, leading to widespread inflammation, tissue damage, and organ failure [1]. Despite advances in critical care medicine, sepsis remains a significant cause of mortality and morbidity, placing a substantial burden on health care systems worldwide [2,3]. In 2017, there were approximately 48.9 million cases of sepsis globally, resulting in around 11 million sepsis-related deaths, constituting 19.7% of all deaths worldwide [4].

The liver, essential for metabolic homeostasis and detoxification, plays a crucial role in sepsis [5]. Sepsis-associated liver injury (SALI) exacerbates the patient’s condition by impairing protein synthesis, disrupting glucose metabolism, and leading to the accumulation of toxic substances [6]. The pathophysiology of SALI involves a complex interplay of inflammatory cytokines, oxidative stress, and mitochondrial dysfunction, which complicates clinical management [7]. Early identification and intervention are critical for improving patient outcomes; however, the nonspecific nature of early clinical signs and the heterogeneity of sepsis make timely diagnosis challenging. Conventional diagnostic tools often fall short, highlighting the need for more sophisticated approaches. Predictive modeling using machine learning (ML) offers a promising solution, as ML can analyze vast amounts of clinical data to uncover hidden patterns and predict adverse outcomes with high precision.

The application of ML in health care, particularly for predicting and diagnosing complex conditions such as sepsis, shows tremendous potential. Traditional ML models, such as neural networks and ensemble methods, can achieve high accuracy but often function as “black boxes,” offering little insight into their decision-making processes. This lack of transparency hinders clinical adoption, as health care providers must be able to understand and trust these tools. To address this, explainable machine learning has emerged, offering models that not only perform well but also provide clear, interpretable explanations for their predictions. Explainable machine learning techniques—such as feature importance scoring, decision trees, and attention mechanisms—reveal the factors driving model decisions, enabling health care professionals to validate the model’s reasoning, gain insights into disease mechanisms, and make informed decisions based on the model’s output. Additionally, advanced ensemble techniques, such as stacking models, are being utilized to further enhance predictive performance. Stacking involves training multiple base models along with a meta-model that integrates their predictions, resulting in a robust and accurate predictive system. This combination of explainability and enhanced performance through stacked models holds great promise for the clinical integration of ML tools.

In this study, we aim to develop an explainable machine learning model, incorporating stacking techniques, to predict the occurrence of liver injury in patients with sepsis. Leveraging a comprehensive clinical database, our goal is to create a model that not only provides accurate predictions but also offers insights into the factors contributing to SALI. This approach ensures that health care professionals can understand and trust the predictions, facilitating better decision-making and personalized patient care. We will detail the methodology used, including data preprocessing, feature selection, model development, and the explainability techniques used to interpret the model’s predictions. By demonstrating the power of explainable ML models and the enhanced performance of stacking approaches, we aim to improve patient outcomes through early and informed interventions.

## Methods

### Study Design

This study was conducted and reported in accordance with the TRIPOD+AI (Transparent Reporting of a Multivariable Prediction Model for Individual Prognosis or Diagnosis, Extended for Artificial Intelligence) guidelines to ensure clarity and transparency in the development and validation of the prediction model [8]. The TRIPOD+AI checklist was completed and is provided as Multimedia Appendix 1 for reference.

### Data Sources

This study utilized 2 large critical care databases: the Medical Information Mart for Intensive Care IV (MIMIC-IV version 2.0) and the eICU-Collaborative Research Database (eICU-CRD, version 2.0). MIMIC-IV is an openly available dataset developed by the Massachusetts Institute of Technology (MIT) Lab for Computational Physiology, containing comprehensive clinical data from patients admitted to intensive care units (ICUs) at the Beth Israel Deaconess Medical Center [9]. The eICU-CRD is a multicenter ICU database that includes data from numerous hospitals across the United States, enhancing the generalizability and robustness of the study findings [10]. Data were deidentified for privacy protection and extracted using structured query language with PostgreSQL 14.0 (PostgreSQL Global Development Group), as described in previous studies [11]. The study adhered to the Reporting of Studies Conducted Using Observational Routinely Collected Health Data (RECORD) statement.

The exclusion criteria were as follows: (1) patients with missing or incomplete key clinical data, (2) patients with ICU stays shorter than 24 hours, and (3) readmissions during the same hospital stay (only the first admission was considered).

### Participants

The inclusion criteria were as follows: (1) patients diagnosed with sepsis according to the Sepsis-3.0 diagnostic criteria [1]; (2) adults over 18 years admitted to the ICU for the first time; (3) no history of chronic liver disease, including chronic viral hepatitis, alcoholic liver disease, liver cancer, biliary system diseases, etc; (4) ICU stays longer than 1 day; and (5) complete records of international normalized ratio (INR) and total bilirubin (TB) on the first day of ICU admission, with follow-up INR and TB within the subsequent week.

### Data Preparation

Data preprocessing involved the following steps: data cleaning, handling missing values, normalization, and feature selection. Briefly, data from the MIMIC-IV dataset (N=8834) were randomly split into training (n=6183, 70%) and internal validation (n=2651, 30%) sets. Data extracted from the eICU-CRD database (N=4236) served as an external validation set, ensuring a robust evaluation of model generalizability across different ICU populations. Missing values were addressed using multiple imputation techniques, while outliers were identified and processed using statistical methods or domain knowledge to ensure data quality and model stability. Important features relevant to the target variable were selected based on domain knowledge and data analysis to enhance the predictive and generalization capabilities of the models. Numerical features were standardized or normalized to eliminate differences in scales among features. Categorical features were dummy encoded to transform them into numerical features suitable for the models. We employed 10-fold cross-validation to assess model performance, where the training dataset was divided into 10 subsets. In each iteration, 9 subsets were used for training, and the remaining subset was used for validation.

Continuous measures were described by the median and quartiles (25th percentile-75th percentile) because the data did not conform to a normal distribution. Nonparametric tests (rank sum test) were used for comparisons between groups.

### Outcome

Given the absence of a gold standard for diagnosing SALI, we defined it based on criteria from previous studies: (1) INR and TB within normal ranges during the first 24 hours of ICU admission; and (2) the development of INR>1.5 and TB>2 within 24 hours to 1 week after ICU admission. This binary outcome was clinically significant, representing patients at high risk of liver dysfunction during sepsis. The outcome was consistently assessed across both the internal and external validation datasets to ensure reliability and comparability.

To ensure consistency and reliability, the outcome was assessed uniformly across both the internal and external validation datasets, following predefined clinical criteria. This consistency minimizes potential biases and enhances the comparability of results. To mitigate the risk of overfitting during model development, several strategies were applied. First, feature selection was conducted using a combination of domain expertise and data-driven approaches, reducing the initial pool of predictors to 42 clinically and statistically relevant variables. Second, hyperparameter tuning was performed using grid search combined with 10-fold cross-validation to optimize parameter selection and assess model performance on unseen subsets of data. Finally, the model’s generalizability and robustness were independently evaluated through external validation using the eICU-CRD dataset. These measures ensured that the final model provided accurate and reliable predictions while minimizing the risk of overfitting.

### Predictors

Demographic data, including age, weight, and gender, were collected. The comorbidity history was documented for conditions such as myocardial infarction, congestive heart failure, peripheral vascular disease (PVD), cerebrovascular disease, chronic pulmonary disease, peptic ulcer disease, paraplegia, chronic kidney disease, malignant neoplasms, and metastatic solid tumors. The initial vital signs recorded upon ICU admission were heart rate, respiratory rate, systolic and diastolic blood pressure, mean arterial pressure, and oxygen saturation. Laboratory data within the first 72 hours of ICU admission included measurements of white blood cells; neutrophils; lymphocytes; monocytes; eosinophils; basophils; red blood cells; platelets; hematocrit; hemoglobin; mean corpuscular hemoglobin; mean corpuscular hemoglobin concentration; mean corpuscular volume; red cell distribution width; alanine transaminase; aspartate transaminase; alkaline phosphatase; albumin; TB; blood urea nitrogen; creatinine; prothrombin time (PT); INR; and levels of sodium, potassium, calcium, chloride, bicarbonate, anion gap, glucose, and lactate. Interventions noted included continuous renal replacement therapy, mechanical ventilation, and vasopressor administration (including dobutamine, dopamine, epinephrine, norepinephrine, and phenylephrine).

### Sample Size

**Figure 1:**
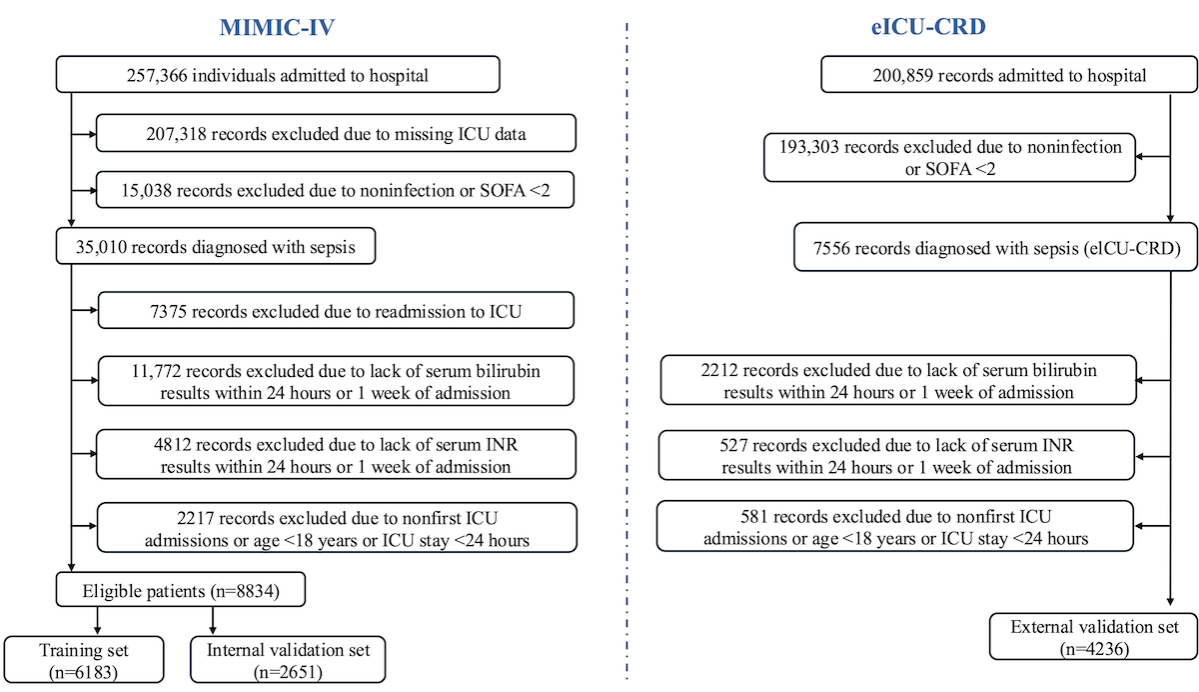
Study design and patient selection process for predicting sepsis-associated liver injury. This figure illustrates the study design and patient selection process used to develop and validate a machine learning model for predicting sepsis-associated liver injury (SALI) in critically ill patients. The study utilized data from 2 critical care databases: Medical Information Mart for Intensive Care IV (MIMIC-IV; n=8834), covering data from 2008 to 2019, and eICU-CRD (n=4236), covering data from 2014 to 2015. Patients were diagnosed with sepsis according to the Sepsis-3.0 criteria and were selected based on predefined inclusion and exclusion criteria, such as missing data or ICU stays of less than 24 hours. The study aimed to identify predictive factors for SALI using machine learning models and to validate these findings using both internal and external datasets. eICU-CRD: eICU-Collaborative Research Database; ICU: intensive care unit; INR: international normalized ratio; SOFA: Sepsis-Related Organ Failure Assessment.

The study cohort was derived from 2 large critical care databases: MIMIC-IV and eICU-CRD (Figure 1). A total of 267,366 records were initially screened in MIMIC-IV, of which 20,731 records were excluded due to the absence of ICU data, 153,805 records were excluded for not meeting the infection or Sepsis-Related Organ Failure Assessment (SOFA)≥2 criteria, and 35,010 records did not have a confirmed sepsis diagnosis. Further exclusions included 7315 records due to readmission to the ICU and 11,772 records for missing serum bilirubin or INR measurements within 24 hours of ICU admission or during the subsequent week. Finally, 8834 eligible patients from MIMIC-IV were included, with 6183 (70%) allocated to the training set and 2651 (30%) to the internal validation set.

Similarly, in the eICU-CRD database, 200,859 records were initially screened. After excluding 193,093 records for not meeting infection or SOFA≥2 criteria, 7566 sepsis cases were identified. Among these, 2212 records were excluded due to missing bilirubin or INR measurements within 24 hours of admission or during the subsequent week, and 551 records were excluded due to ICU stays shorter than 24 hours or duplicate admissions. A total of 4236 eligible patients were included in the external validation set. The number of positive events was as follows: training set: 428 (10.10%); internal validation set: 165 (3.90%); and external validation set: 648 (15.30%).

According to the sample size calculation method [12,13], model development must adhere to the principle of at least 10 events per variable, while also considering the event rate and total sample size. The current model includes 42 predictors, and based on the formula *n*_event_≥*k* × 10 (where *k* is the number of predictors), at least 420 events are required. Considering the event rate *p*=(428/6183)≈6.92% and the formula *n*≥[(*k* × 10)/*p*], the total required sample size is approximately 6070. The actual sample size (training set n=6183 and event count=428) meets the requirements of the events per variable principle and its derived rules, ensuring adequacy for model development. The external validation set has a higher number of positive events and event rate, which also meets the sample size requirements for events per variable in model development.

### Missing Data

Datasets with more than 30% missing values were excluded from the analysis. Missing data were handled using multiple imputation via the “mice” package in R (R Foundation for Statistical Computing). Five imputed datasets were generated to account for uncertainty due to missingness. All 42 predictors and the outcome variable (SALI) were included in the imputation process to ensure consistency and avoid bias in downstream analyses. For continuous variables, predictive mean matching was used to preserve the observed data distribution, while categorical variables were imputed using logistic regression. The imputation process involved 10 iterations to ensure convergence, and pooled estimates of model coefficients and confidence intervals were obtained using the Rubin rules. This approach ensures robust handling of missing data by accounting for the uncertainty associated with missing values while maintaining the integrity of the predictive model.

### Analytical Methods

Nine different ML models were used to predict patient outcomes: decision tree, random forest (RF), extreme gradient boosting (XGBoost), support vector machine, elastic net, multilayer perceptron, light gradient boosting machine (LightGBM), *k*-nearest neighbors, and logistic regression. The models were implemented using R Studio and popular ML packages such as “caret,” “xgboost,” and “lightgbm.” Hyperparameter tuning was performed using grid search and cross-validation to optimize model performance.

To enhance predictive performance, a stacking ensemble method was used. In this approach, predictions from the base models (decision tree, RF, XGBoost, support vector machine, etc) served as input features for a meta-model, typically logistic regression, which provided the final prediction. This method leverages the strengths of individual models, potentially improving overall prediction accuracy and robustness.

### Model Performance Evaluation

#### Assessment of Discrimination, Calibration, and Clinical Utility of ML Models for SALI Prediction

We assessed the performance of the ML models used to predict SALI based on discrimination, calibration, and clinical usefulness. Various classification measures, including receiver operating characteristic (ROC) curve analysis, precision-recall (PR) curve analysis, Brier score, and decision curve analysis (DCA), were used to evaluate the models’ discriminative ability, calibration, and net benefit at different threshold probabilities.

#### Discrimination

Discrimination refers to the model’s ability to distinguish between patients who develop SALI and those who do not. The area under the ROC curve (ROC-AUC) was the primary metric for evaluating discrimination, with values ranging from 0.5 (no discrimination) to 1.0 (perfect discrimination). A higher ROC-AUC value indicates a better ability to discriminate between positive and negative cases. Additionally, the PR area under the curve (PR-AUC) was calculated to account for class imbalance, quantifying the trade-off between sensitivity (recall) and positive predictive value (precision). PR-AUC is particularly valuable when the classes are imbalanced, as is the case with SALI prediction. Secondary discrimination metrics included accuracy, balanced accuracy, sensitivity (recall), specificity, *F*_1_-score (the harmonic mean of precision and recall), Matthew correlation coefficient [14], and the Jaccard index (J-index) [15].

#### Calibration

Calibration assesses the agreement between predicted probabilities and observed outcomes [16]. Calibration plots were used, with predicted probabilities plotted against the observed event rates in deciles of predicted risk. The 45° line represents perfect calibration, where predicted probabilities perfectly match the observed outcomes. We also used the Brier score to quantify calibration, which calculates the mean-squared difference between predicted probabilities and actual outcomes [17]. Lower Brier scores indicate better calibration of the model.

In addition to these, positive predictive value, negative predictive value, and detection prevalence were used as additional calibration metrics. These metrics were assessed to further evaluate the consistency of the model’s predictions across different deciles and to analyze whether the model overestimated or underestimated risk in specific subgroups.

#### Clinical Utility

Clinical utility was assessed through DCA, which evaluates the net benefit of using the model at a range of threshold probabilities. Net benefit was calculated as the proportion of true positives, adjusted for the harm of false positives, relative to the reference strategies of “treat all” and “treat none.” A higher net benefit indicates that the model provides more clinical value, facilitating more informed decision-making in the clinical setting [18,19].

### Model Selection in the Stacking Process

The selection of base models and the meta-model in the stacking framework was guided by both prior research and empirical evaluation during preliminary experiments. Base models were chosen to include a diverse set of ML algorithms, such as tree-based models (LightGBM, RF, XGBoost), linear models (elastic net, logistic regression), and neural networks (multilayer perceptron). This diversity ensures that the stacking framework effectively leverages the complementary strengths of different algorithmic approaches, enhancing predictive power and robustness.

Lasso regression was selected as the meta-model due to its ability to perform variable selection and shrinkage simultaneously, reducing the risk of overfitting while maintaining interpretability. This characteristic makes Lasso regression particularly well-suited for aggregating predictions from multiple base models, especially in high-dimensional settings. During the stacking process, we optimized the hyperparameter (λ) for Lasso regression using cross-validation to balance bias and variance. This approach ensured that the meta-model retained the most informative base model predictions while discarding redundant or less relevant contributions.

### Model Output

To interpret the contributions of each feature in the ML models, Shapley additive explanations (SHAP) values were used. SHAP values provide a unified measure of feature importance by attributing the change in model output to each feature, ensuring consistency and fairness in the interpretation. This method helps to understand the impact of each feature on the model’s predictions, thereby enhancing transparency and trust in the model’s decisions.

### Ethical Considerations

This study is a secondary analysis of publicly available, deidentified patient data from the MIMIC-IV (version 2.0) and the eICU-CRD (version 2.0). The use of MIMIC-IV data has been approved by the MIT Institutional Review Board under protocol number 0403000206. As all data in MIMIC-IV are fully deidentified in compliance with the Health Insurance Portability and Accountability Act (HIPAA) regulations, this study was classified as nonhuman research, and no additional institutional review board approval was required. JL has completed the required Collaborative Institutional Training Initiative (CITI) certification for access to MIMIC-IV and eICU-CRD data (CITI record ID: 64190160). Similarly, the eICU-CRD dataset is accessible under a data use agreement, and all patient data have been deidentified, eliminating the need for additional institutional review board approval. As this study exclusively uses deidentified secondary data, informed consent was waived. No identifiable patient information was accessed, and all analyses were conducted in accordance with MIMIC-IV and eICU-CRD data use policies.

### Software and Tools

All analyses were conducted using the R programming language (version 4.4.1) with R Studio as the integrated development environment. The primary libraries and tools used were mice (version 3.15.0) for multiple imputation, dplyr (version 1.1.4) for data manipulation, caret (version 6.0-94) for ML algorithms, xgboost (version 1.7.8.1) for gradient boosting, and ggplot2 (version 3.5.1) for data visualization.

## Results

### Participants

This study adhered to the TRIPOD+AI guidelines throughout model development and validation to ensure methodological transparency. The completed checklist is provided as Multimedia Appendix 1. According to the inclusion and exclusion criteria, a total of 8834 patients from the MIMIC-IV database and 4236 patients from the eICU-CRD database were included (Figure 1). The MIMIC-IV patients were divided into a training set and an internal validation set in a 7:3 ratio, with 6183 patients in the training set and 2651 patients in the internal validation set. Before analysis, we conducted a correlation analysis on the data from the MIMIC-IV database, focusing on the continuous variables in both the training and internal validation sets. Continuous variables with a correlation coefficient greater than 0.5 were excluded. As a result, 42 independent variables (including both continuous and categorical variables) were included in the subsequent analysis (see Figure S1 in Multimedia Appendix 2). There were no significant statistical differences between the training set and the internal validation set regarding each variable or the incidence of SALI (Table 1). Among all patients, the incidence of SALI was 428 out of 6183 (6.92%) in the training set, 165 out of 2651 (6.22%) in the internal validation set, and 648 out of 4358 (15.30%) in the external validation set (Table 1).

**Table 1:** Baseline characteristics, clinical tests, interventions, and outcomes of patients with sepsis from MIMIC-IV^a^ and eICU-CRD^b^ databases.

Variable		MIMIC-IV database					eICU-CRD dataset
		Training set (n=6183)	Internal validation set (n=2651)	*P* value		External validation set (n=4236)	
Age (years), median (25th to 75th percentile)		69.4 (57.7 to 80.1)	69.1 (57.5 to 80.2)	.81		66.5 (56.0 to 77.0)	
Gender male, n (%)		3489 (56.43)	1442 (54.39)	.08		2289 (52.52)	
Weight (kg), median (25th to 75th percentile)		78.0 (65.6 to 94.1)	78.0 (65.9 to 93.7)	.65		79.0 (64.1 to 98.2)	
**Comorbidity, n (%)**							
	Myocardial infarction	1183 (19.13)	515 (19.43)	.75		248 (5.69)	
	Peptic ulcer disease	174 (2.81)	87 (3.28)	.23		25 (0.57)	
	Congestive heart failure	1852 (29.95)	793 (29.91)	.97		466 (10.69)	
	Peripheral vascular disease	737 (11.92)	342 (12.90)	.20		31 (0.71)	
	Dementia	389 (6.29)	167 (6.30)	.99		126 (2.89)	
	Chronic pulmonary disease	1593 (25.76)	707 (26.67)	.37		490 (11.24)	
	Diabetes	1848 (29.89)	801 (30.22)	.76		709 (16.27)	
	Renal disease	1348 (21.80)	608 (22.93)	.24		2276 (52.23)	
	Malignant cancer	987 (15.96)	407 (15.35)	.47		217 (4.98)	
**Vital signs, median (25th to 75th percentile)**							
	Heart rate (beats per minute)	104 (90 to 119)	105 (92 to 120)	.01^c^		99 (84 to 114)	
	Mean blood pressure (mm Hg)	58 (51 to 84)	58 (51 to 82)	.05		75 (66 to 86)	
	Respiratory rate (breaths per minute)	28 (24 to 32)	28(24 to 32)	.57		20 (17 to 26)	
	Body temperature (°C)	36.9 (36.1 to 37.7)	36.7 (35.9 to 37.6)	.02^c^		36.9 (36.5 to 37.4)	
	Oxygen saturation (%)	97.2 (95.8 to 98.5)	97.2 (95.7 to 98.5)	.52		98.0 (95.0 to 100.0)	
**Laboratory tests, median (25th to 75th percentile)**							
	White blood cell (K/μL)	10.9 (7.5 to 15.5)	11.00 (7.4 to 15.8)	.98		14.1 (9.3 to 19.6)	
	Hemoglobin (g/mL)	11.1 (9.4 to 12.7)	11.1 (9.4 to 12.7)	.34		11.3 (9.5 to 13.0)	
	Platelet (K/μL)	198 (145 to 265)	201 (143 to 266)	.97		216 (153 to 297)	
	Mean corpuscular hemoglobin concentration (g/mL)	32.9 (31.8 to 33.9)	32.8 (31.8 to 33.9)	.21		32.6 (31.6 to 33.6)	
	Mean corpuscular volume (fL)	91 (87 to 96)	91 (87 to 97)	.66		91.0 (86.4 to 95.6)	
	Red cell distribution width (%)	14.3 (13.4 to 15.7)	14.4 (13.4 to 15.7)	.15		15.2 (14.0 to 16.9)	
	Oxygen pressure (mm Hg)	70 (40 to 100)	67 (41 to 97)	.06		91 (69 to 140)	
	Carbon dioxide pressure (mm Hg)	46 (40 to 53)	46 (40 to 53)	.79		39 (32 to 48)	
	Base excess	0 (–2 to 3)	0 (–2 to 3)	.59		0 (–5.5 to 3.9)	
	Lactate (mmol/L)	2.0 (1.3 to 3.3)	2.1 (1.4 to 3.4)	.01^c^		2.0 (1.2 to 3.3)	
	Glucose (mg/dL)	133 (111 to 171)	132 (111 to 173)	.82		142 (109 to 196)	
	Anion gap	16 (14 to 19)	16 (14 to 19)	.39		12 (9 to 15.4)	
	Alanine transaminase (U/L)	23 (15 to 44)	24 (14 to 45)	.76		26 (17 to 46)	
	Alkaline phosphatase (U/L)	77 (58 to 105)	76 (57 to 105)	.32		90 (67 to 128)	
	Total bilirubin (mg/dL)	0.5 (0.3 to 0.8)	0.5 (0.3 to 0.8)	.94		0.6 (0.4 to 0.9)	
	Creatinine (mg/dL)	1.2 (0.8 to 1.8)	1.2 (0.9 to 1.9)	.14		1.3 (0.9 to 2.3)	
	Potassium (mmol/L)	4.1 (3.7 to 4.6)	4.1 (3.8 to 4.7)	.03^c^		4.1 (3.7 to 4.7)	
	Calcium (mg/dL)	8.3 (7.7 to 8.8)	8.2 (7.6 to 8.8)	.15		8.3 (7.7 to 8.9)	
	Chloride (mmol/L)	104 (100 to 108)	104 (100 to 108)	.40		103 (98 to 108)	
	Prothrombin time (seconds)	13.5 (12.2 to 14.9)	13.5 (12.3 to 14.8)	.23		14.1 (12.6 to 15.7)	
	Partial thromboplastin time (seconds)	29.3 (26.2 to 33.8)	29.3 (26.3 to 34.0)	.71		30.7 (27.0 to 35.0)	
	Urine output (mL) (day 1)	1550 (918 to 2375)	1550 (940 to 2450)	.27		2350 (910 to 4962)	
**Intervention** **,** **n (%)**							
	Vasopressor used	2734 (44.22)	1209 (45.61)	.23		1410 (32.35)	
	Continuous renal replacement therapy	293 (4.74)	132 (4.98)	.63		185 (4.25)	
	Ventilation	2482 (40.14)	1073 (40.48)	.77		1996 (45.80)	
SALI^d^ incidence, n (%)		428 (6.92)	165 (6.22)	.23		648 (15.30)	

^a^MIMIC-IV: Medical Information Mart for Intensive Care IV.

^b^eICU-CRD: eICU-Collaborative Research Database.

^c^Statistically significant (*P*<.05).

^d^SALI: sepsis-associated liver injury.

### Model Development: Performance of Individual Machine Learning Models

**Figure 2:**
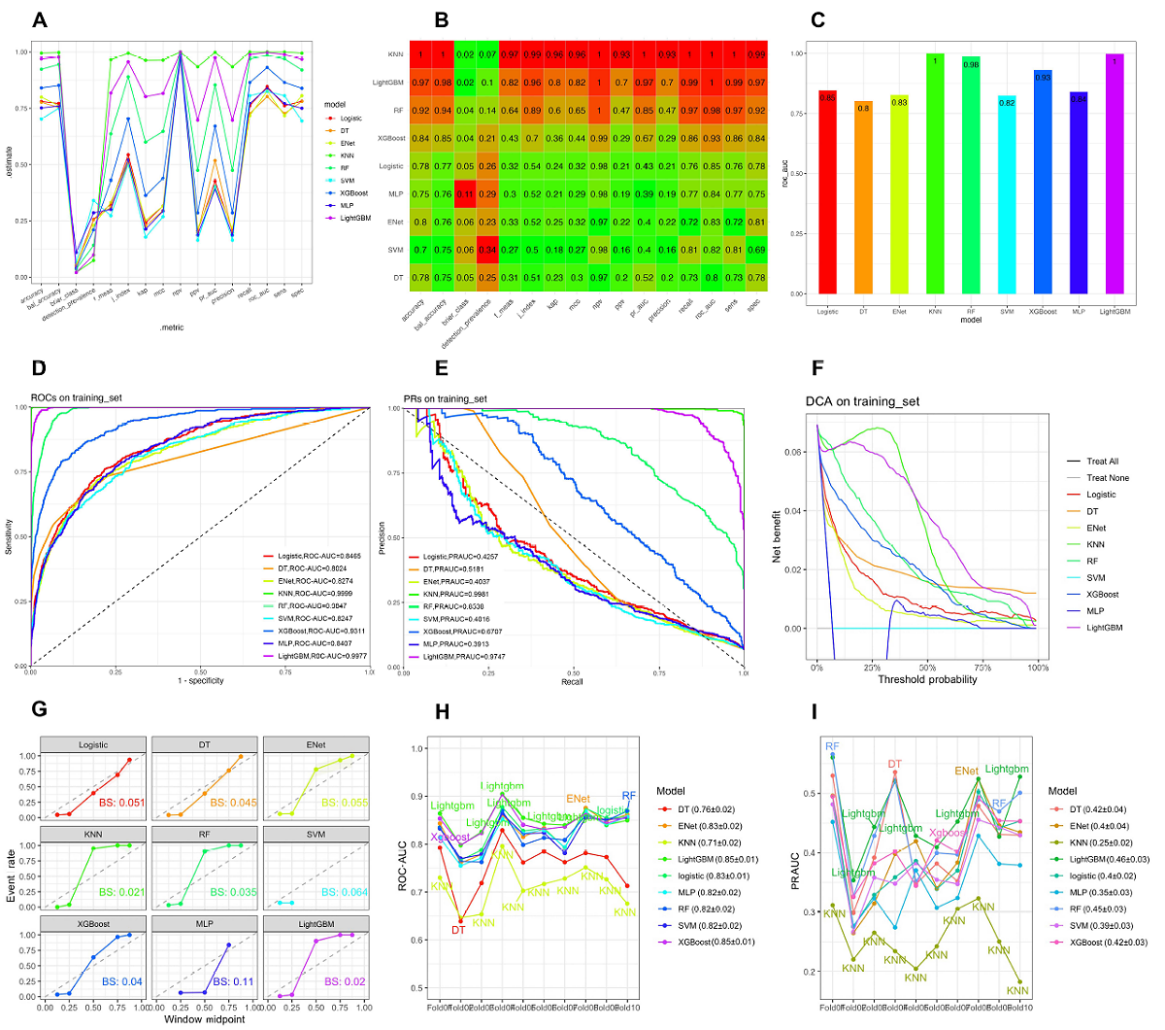
This figure illustrates the performance of 9 machine learning models, including light gradient boosting machine (LightGBM), extreme gradient boosting (XGBoost), and random forest (RF), in predicting sepsis-associated liver injury (SALI) in patients with sepsis. Performance is evaluated using area under the receiver operating characteristic curve (ROC-AUC), precision-recall (PR)–AUC, calibration curves, and decision curve analysis (DCA), ensuring robustness and clinical applicability. (A) Line plot of model performance across multiple metrics, demonstrating the consistency and variability of each model. (B) Heat map of model performance, with color gradients representing the relative strengths and weaknesses across metrics. (C) Bar chart comparing the overall performance scores of each model, highlighting the best-performing algorithms. (D) ROC curves of the models on the training set, indicating discriminatory power via ROC-AUC values. (E) PR curves on the training set, showing model performance in distinguishing positive cases. (F) DCA on the training set, illustrating the net benefit of each model at different decision thresholds. (G) Calibration plots depicting the Brier scores (BSs) of each model, reflecting the accuracy of predicted probabilities. (H) ROC-AUC values across cross-validation folds, indicating model stability and performance consistency. (I) PR-AUC values across cross-validation folds, demonstrating model effectiveness in identifying positive cases across different data subsets. DT: decision tree; ENet: elastic net; KNN: k-nearest neighbors; MLP: multilayer perceptron; SVM: support vector machine.

The model optimization framework combined random grid search (with 20 hyperparameter combinations and 10-fold cross-validation) and Bayesian optimization (with 50 iterations, initialized by 10 evaluations), selecting optimal configurations based on ROC-AUC performance (see Figure S2 in Multimedia Appendix 2). Evaluations of the training set across multiple metrics—such as ROC/PR curves, calibration plots, and decision curve analyses (Figure 2A-G; high resolution version available in Multimedia Appendix 3)—consistently identified LightGBM, XGBoost, and RF as the top performers.

These models exhibited minimal variance in cross-validation stability, as shown by the tightly clustered ROC-AUC and PR-AUC distributions (Figure 2H, I), in contrast to the broader fluctuations observed in other algorithms. Specifically, in the training set, LightGBM achieved the highest ROC-AUC of 0.9977, followed by XGBoost (ROC-AUC 0.9311) and RF (ROC-AUC 0.9847), demonstrating their robust performance across evaluation metrics.

**Figure 3:**
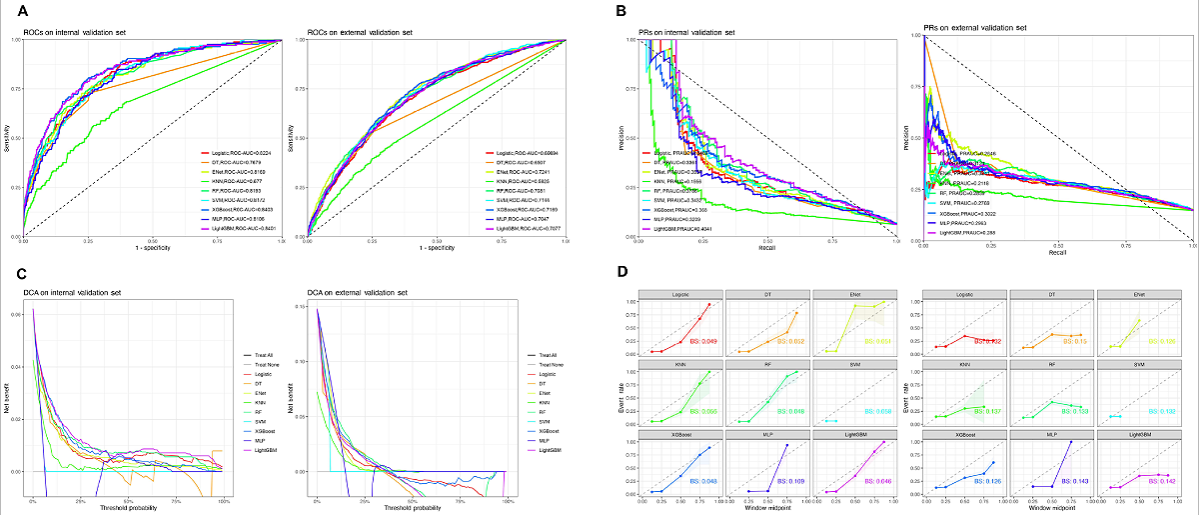
Comparative evaluation of machine learning models on internal and external validation sets. The figure presents receiver operating characteristic (ROC) curves, precision-recall (PR) curves, decision curve analysis, and calibration plots for model validation. The internal validation set was derived from the Medical Information Mart for Intensive Care IV (MIMIC-IV) database, while the external validation set used eICU–Collaborative Research Database (eICU-CRD) data to assess model generalizability. (A) ROC curves for models on the internal and external validation sets, showing sensitivity versus 1 – specificity with corresponding ROC–area under the curve (AUC) values, which assess the models’ ability to distinguish between classes. (B) PR curves on the internal and external validation sets, displaying precision versus recall to evaluate model performance in identifying positive cases, particularly in imbalanced datasets. (C) Decision curve analysis on the internal and external validation sets, illustrating the clinical utility of the models by comparing the net benefit across different threshold probabilities. (D) Calibration plots for models on the internal and external validation sets, with Brier scores (BSs) indicating the alignment between predicted probabilities and actual outcomes, thereby assessing the models’ calibration quality. Enet: elastic net; KNN: k-nearest neighbors; LightGBM: light gradient boosting machine; MLP: multilayer perceptron; RF: random forest; SVM: support vector machine; XGBoost: extreme gradient boosting.

Internal (MIMIC-IV holdout) and external (eICU-CRD) validations confirmed the generalizability of LightGBM and XGBoost, with ROC/PR curves (Figure 3A, B; high resolution version available in Multimedia Appendix 3) showcasing their consistent discriminative power across datasets. In the internal validation, LightGBM (ROC-AUC 0.8401) and XGBoost (ROC-AUC 0.8403) outperformed other models, with RF demonstrating competitive performance (ROC-AUC 0.8193). In the external validation set, LightGBM (ROC-AUC 0.7077), XGBoost (ROC-AUC 0.7169), and RF (ROC-AUC 0.7081) maintained strong performance, although there was a slight decrease in ROC-AUC compared with the training set, highlighting the models’ generalizability.

DCA further evaluated the clinical utility of these models across different threshold probabilities (Figures 2F and 3C), demonstrating that LightGBM and XGBoost provided superior net benefits at lower thresholds in both the internal and external validation sets. Calibration fidelity was further assessed through Brier score comparisons (Figure 2G and 3D), where LightGBM and XGBoost demonstrated superior calibration across both internal and external datasets, reflecting better alignment between predicted probabilities and observed event rates.

### Model Updating: Stacking Model Development and Performance

To enhance prediction performance, we combined the strengths of XGBoost, LightGBM, and RF into a stacking ensemble model. The stacking model aggregated the outputs of these individual models, with Lasso regression serving as the meta-model to combine the predictions. The performance of the stacking model was evaluated across the training set, internal validation set, and external validation set, showing superior performance compared with the individual models. Table 2 demonstrates the calibration performance of the model in different datasets.

**Figure 4:**
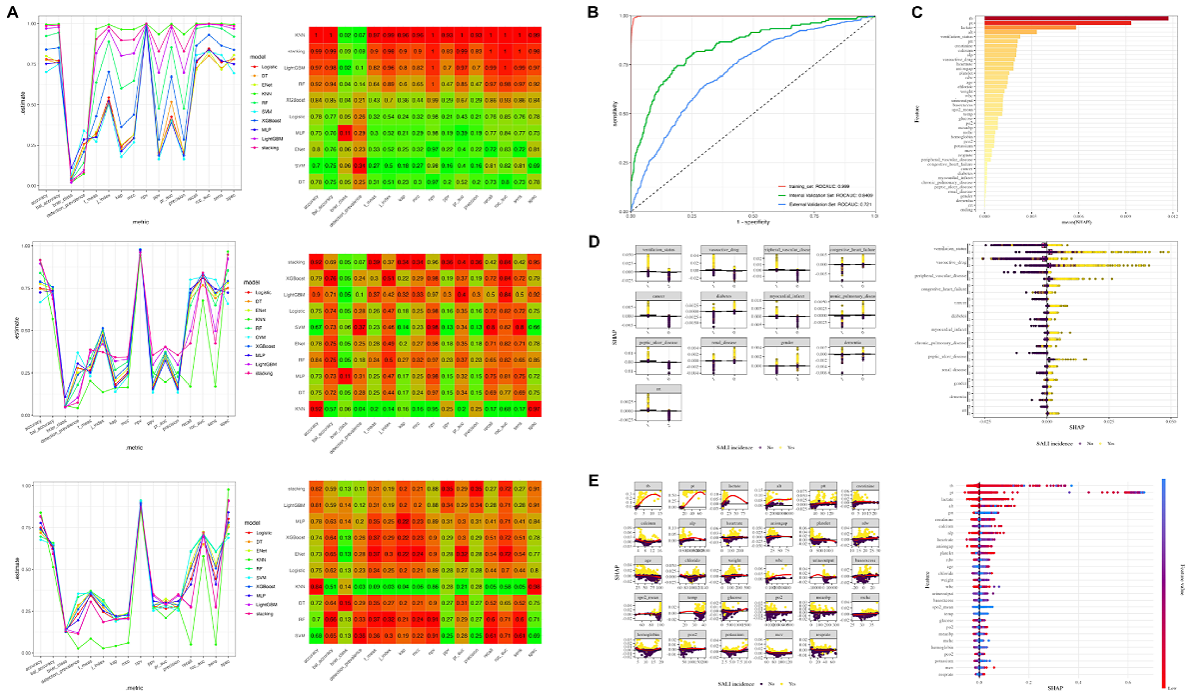
Stacking ensemble model for predicting sepsis-associated liver injury: performance and Shapley additive explanations (SHAP) analysis. This figure compares the stacking model’s performance against individual base models, demonstrating its superior predictive capability. Receiver operating characteristic (ROC) curves and SHAP analysis highlight key predictive features, confirming their clinical significance. (A) Line plots and heat maps comparing model performance across various metrics. (B) ROC curves for the stacking model, showing its performance on the training set, internal validation set, and external validation set. (C) Bar plot of mean absolute SHAP values. (D) SHAP summary plots and SHAP dependence plots for categorical variables of the stacking model, showing the distribution of SHAP values for key features. (E) SHAP summary plots and SHAP dependence plots for continuous variables of the stacking model. ALP: alkaline phosphatase; ALT: alanine transaminase; BP: blood pressure; MCHC: mean corpuscular hemoglobin concentration; MCV: mean corpuscular volume; PCO2: partial pressure of carbon dioxide; PO2: partial pressure of oxygen; PT: prothrombin time; PTT: partial thromboplastin time; RDW: red cell distribution width; RRT: renal replacement therapy; SpO2: peripheral capillary oxygen saturation; TB: total bilirubin; WBC: white blood cell.

A heat map illustrates the performance of the stacking model and all other models across the training set, internal validation set, and external validation set, using various evaluation metrics (Figure 4A; high resolution version available in Multimedia Appendix 3). A line chart further highlights the differences in model performance on these metrics (Figure 4B). In the training set, the stacking model achieved an ROC-AUC of 0.999, substantially outperforming the individual models. In the internal validation set, the stacking model maintained strong performance with an ROC-AUC of 0.841, comparable to LightGBM and XGBoost, but with improved consistency and reduced variance. In the external validation set, the stacking model achieved an ROC-AUC of 0.721, outperforming most individual models and further confirming its generalizability (Figure 4B).

SHAP values quantify the contribution of each feature to the model’s predictions, accounting for both the feature’s importance and how different values of the feature influence the model’s output. The SHAP analysis of both individual high-performance models (XGBoost, LightGBM, and RF) and the stacked ensemble consistently identified TB, lactate, PT, and alanine transaminase as the most influential continuous predictors for SALI in patients with sepsis. Additionally, categorical variables such as ventilation status, vasoactive drug use, and PVD demonstrated comparable clinical importance (see Figures S3-S5 in Multimedia Appendix 2 and 4C-E). In XGBoost-derived visualizations, mean SHAP bar plots and summary plots highlighted the dominant contributions of these features (see Figure S5A in Multimedia Appendix 2), while SHAP dependence plots with trend lines quantitatively mapped their nonlinear impacts across value ranges (see Figure S5D in Multimedia Appendix 2 and Figure 4D). The broad distributions in the box plots (see Figure S5C, E in Multimedia Appendix 2) and consistent pattern replication in the stacking model’s SHAP visualizations (Figure 4C-E) collectively confirm the robustness of these biomarkers. Notably, ventilation status emerged as the strongest categorical determinant across all model architectures, paralleling the pathophysiological weight of TB and lactate in the analysis of continuous parameters. This multimodel interpretability framework converged on a stable set of prognostic indicators, aligning patterns of feature importance with clinical markers of hepatobiliary and hemodynamic dysfunction.

**Table 2:** Performance metrics before and after calibration of stacking models for predicting sepsis-associated liver injury.

Dataset and calibration		Observed	Expected	Observed-to-expected ratio	C-index
**Training set**					0.99565
	Uncalibrated	428	463.8876	0.9226372	
	Calibrated	428	428	1	
**Internal validation set**					0.83793
	Uncalibrated	165	179.5138	0.919143	
	Calibrated	165	335.7046	0.4515035	
**External validation set**					0.720543
	Uncalibrated	648	385.4175	1.681293	
	Calibrated	648	648	1	

## Discussion

### Principal Findings

Systemic dysregulation in patients with sepsis, leading to multiorgan failure, is the primary cause of mortality. Because of the heterogeneity in etiology, host response, and organ environments, consistently effective methods to save patients’ lives remain elusive [20]. It is now widely believed that precise stratification and early identification of high-risk patients are among the most feasible approaches to reducing sepsis-related mortality. In this study, we developed and evaluated a stacking ensemble model to predict SALI in critically ill patients, using data from 2 databases (MIMIC-IV and eICU-CRD). Our results demonstrate that the stacking model, which leverages the strengths of individual ML algorithms, achieved superior predictive performance in both internal and external validation settings. Notably, the models used in the stacking ensemble assigned high importance to features such as TB, PT, lactate levels, ventilation status, vasoactive drug use, and PVD.

### Comparison With Prior Work

Sepsis is the second leading cause of cholestasis in patients presenting with clinical jaundice [21], primarily associated with gram-negative bacterial infections. Even extrahepatic infections are frequently accompanied by elevated markers of cholestasis [22]. In the ICU, 11%-36% of patients exhibit cholestasis [23], with associated mortality rates ranging from 27% to 48% [24,25]. In the liver, critical transmembrane transport systems responsible for the uptake of endogenous and exogenous substances from sinusoidal blood include organic cation transporters, organic anion transporters, organic anion transporting polypeptides, and sodium taurocholate cotransporting polypeptide. In sepsis, inflammation not only reduces the anchoring of these transporters to the membrane [26], but also a significant number of drugs—acting as competitive inhibitors—can impair their function, leading to severe metabolic disturbances [27,28]. In our ML model, TB showed a high predictive contribution, consistent with previous studies demonstrating a close relationship between cholestasis and liver dysfunction. An elevation in TB indicates the presence and severity of cholestasis, underscoring the importance of monitoring liver function and detecting cholestasis early in ICU patients, particularly those with sepsis.

PT is a commonly used laboratory indicator for assessing blood coagulation function, primarily evaluating the extrinsic coagulation pathway. PT-INR on day 1 has been identified as the most accurate independent prognostic predictor in patients with acute liver injury [29]. However, it is important to note that while both PT and activated partial thromboplastin time are used to diagnose coagulation disorders, activated partial thromboplastin time was assigned a very low contribution in our model, with a narrow SHAP value distribution. Considering the common coagulation pathway, we believe that PT reflects factors beyond the coagulation cascade model. Neither PT nor activated partial thromboplastin time can fully capture the critical roles of the antithrombotic and fibrinolytic systems, but the key difference is that PT can indicate the liver’s synthetic capacity [30]. The higher the PT, the greater the corresponding SHAP value, reflecting the model’s contribution related to impaired hepatic synthetic function. Future research should further investigate the potential of combining PT with other biomarkers to develop a more accurate prognostic model, ultimately improving treatment outcomes for patients with acute liver injury.

In the body, lactate serves not only as an energy source but also as a signaling molecule, acting through protein lactylation [31,32]. The liver has the highest lactate clearance rate among all organs, accounting for approximately 70% of total systemic clearance [32,33]. Lactate can serve as a prognostic indicator and is associated with short-term mortality in critically ill patients with liver cirrhosis [34]. Additionally, lactate and its metabolism are linked to the progression of liver fibrosis, nonalcoholic fatty liver disease, and hepatocellular carcinoma [35-37]. In patients with sepsis, lactate is a crucial prognostic marker, with elevated levels closely linked to disease severity and mortality [38]. According to the Sepsis-3 guidelines [39], septic shock should be clinically defined if serum lactate levels remain above 2 mmol/L despite adequate fluid resuscitation. Similarly, elevated lactate levels are closely associated with the number of organ failures and mortality in patients with liver cirrhosis. Both the initial lactate level at admission and subsequent lactate clearance serve as important predictors of short- and long-term mortality in patients with cirrhosis [34,40]. The American Association for the Study of Liver Diseases [41] has highlighted that in patients with acute liver failure, an elevated lactate level (>4 mmol/L) is an independent risk factor for poor prognosis. In patients with chronic liver disease, lactate levels are strongly correlated with in-hospital mortality. Moreover, elevated lactate levels have been identified as an independent predictor of 6-month mortality in patients with hepatitis B–related decompensated cirrhosis [42]. In our model, lactate exhibited a high SHAP value distribution, indicating the need to focus on lactate’s dual role in sepsis and severe liver diseases to develop a more comprehensive and precise monitoring system.

Previous studies have reported the relationship between mechanical ventilation and liver injury. In models of acute respiratory distress syndrome, the use of a high positive end-expiratory pressure open lung strategy has been shown to induce neutrophil infiltration in the liver [43]. High positive end-expiratory pressure during lung recruitment maneuvers may lead to a more pronounced hepatic inflammatory response and elevated liver-related enzyme levels [44]. The association between mechanical ventilation and liver injury has also been reported in patients with COVID-19: abnormal liver enzyme levels in patients with COVID-19 are closely related to the need for mechanical ventilation and the severity of the disease [45]. Liver dysfunction is common in patients with COVID-19 and other respiratory viral infections, and it is associated with poor clinical outcomes [46]. Mechanical ventilation can increase the levels of cytokines in the lungs and blood, leading to systemic leukopenia and damage to the liver, intestine, and kidneys. Furthermore, the interaction between mechanical ventilation and endotoxemia can activate proinflammatory pathways in the lungs and systemic circulation, resulting in dysfunction and/or injury of distal organs [47,48]. Our model supports the relationship between mechanical ventilation and liver injury, as the feature of mechanical ventilation contributed a higher SHAP value, suggesting that clinical practice should consider the potential liver damage caused by mechanical ventilation.

It is now believed that the impact of vasoactive drugs on SALI is primarily achieved through their regulation of hepatic hemodynamics and the balance of oxygen supply and demand. Some researchers have pointed out that an increased hepatic artery resistance index (HARI) and a reduced peak portal vein velocity are predictors of poor prognosis in patients with acute liver injury [49]. Vasoconstrictors, by increasing hepatic perfusion pressure and improving liver blood flow, may potentially enhance prognosis. However, excessive use of these drugs may lead to hepatic ischemia-reperfusion injury, exacerbating hepatocellular damage [50]. Vasodilators are primarily used to lower portal vein pressure and improve liver blood flow; however, while these drugs can reduce vascular resistance, they may also lead to systemic hypotension, further reducing hepatic perfusion [51]. Our findings suggest that during the treatment of SALI, strict monitoring and adjustment of liver function may be necessary when using vasoactive drugs to minimize potential adverse effects on patient outcomes.

Patients with PVD often experience endothelial dysfunction, which may lead to reduced hepatic blood flow and ischemia-reperfusion injury. Hepatic microcirculatory disturbances can result in hepatocyte hypoxia and necrosis, thereby exacerbating liver injury [52]. Additionally, patients with PVD exhibit elevated systemic inflammation, with Kupffer cells and neutrophils in the liver becoming activated in this inflammatory environment, releasing more cytokines and oxidants, which further damage hepatocytes [53,54]. Patients with PVD also frequently have abnormalities in the coagulation system, potentially leading to microvascular thrombosis in the liver, further worsening ischemia and liver damage [55,56]. Our model emphasizes the role of PVD in predicting liver injury, suggesting that PVD may not only be a comorbid condition but also a key contributor to hepatic dysfunction. For patients with PVD, careful monitoring and management of liver function should be considered to mitigate potential liver damage.

### Strengths and Limitations

This study has several notable strengths. First, we leveraged multicenter data from 2 large ICU databases (MIMIC-IV and eICU-CRD), encompassing 13,070 critically ill patients, to enhance generalizability. The use of explainable ML techniques provided clinically interpretable insights into nonlinear relationships between predictors and outcomes. The stacking ensemble framework further improved predictive robustness by integrating diverse algorithms (LightGBM, XGBoost, and RF), achieving state-of-the-art performance. Multiple performance metrics, including ROC-AUC, PR-AUC, Brier score, and DCA, were used to evaluate the model’s predictive accuracy and clinical utility. Additionally, adherence to TRIPOD+AI guidelines ensured methodological rigor and transparency.

However, limitations must be acknowledged. First, the retrospective design introduces potential selection bias and unmeasured confounders, such as genetic factors and drug interactions, which may influence the risk of SALI. Future prospective studies should address these factors. Second, the lower performance in the external validation cohort (ROC-AUC 0.721) is likely due to interhospital variability in data collection and patient populations. Expanding the dataset to include more diverse hospitals and regions could improve generalizability. Third, the definition of SALI based on laboratory thresholds rather than histological confirmation may have misclassified subclinical liver injury. Incorporating histological evaluation or advanced imaging in future studies could refine this definition. Fourth, the US-based ICU data limits generalizability to other health care settings, and international cohort validation is needed. Finally, the exclusion of real-time dynamic variables due to data quality constraints may affect predictive accuracy. Therefore, future studies should integrate real-time data streams to enhance model performance in clinical workflows.

### Conclusions

The stacking ensemble model developed in this study yields accurate and robust predictions of SALI in patients with sepsis, demonstrating potential clinical utility for early intervention and personalized treatment strategies.

## Data Availability

All intermediate data can be obtained from the authors upon reasonable request, and the related code is available on GitHub [57].

## Appendix

Multimedia Appendix 1 TRIPOD+AI checklist.

Multimedia Appendix 2 Additional charts.

Multimedia Appendix 3 Higher resolution versions of Figures 2-4C.

## Abbreviations

AUC: area under the curve
CITI: Collaborative Institutional Training Initiative
DCA: decision curve analysis
eICU-CRD: eICU-Collaborative Research Database
HARI: hepatic artery resistance index
HIPAA: Health Insurance Portability and Accountability Act
ICU: intensive care unit
INR: international normalized ratio
LightGBM: light gradient boosting machine
MIMIC-IV: Medical Information Mart for Intensive Care IV
MIT: Massachusetts Institute of Technology
ML: machine learning
PR: precision-recall
PT: prothrombin time
PVD: peripheral vascular disease
RECORD: Reporting of Studies Conducted Using Observational Routinely Collected Health Data
RF: random forest
ROC: receiver operating characteristic
ROC-AUC: area under the ROC curve
SALI: sepsis-associated liver injury
SHAP: Shapley additive explanations
SOFA: Sepsis-Related Organ Failure Assessment
TB: total bilirubin
TRIPOD+AI: Transparent Reporting of a Multivariable Prediction Model for Individual Prognosis or Diagnosis, Extended for Artificial Intelligence
XGBoost: extreme gradient boosting
