# Supplementary material for: Supervised Machine Learning Models for Predicting Sepsis-Associated Liver Injury in Patients With Sepsis: Development and Validation Study Based on a Multicenter Cohort Study
Source: J Med Internet Res. 2025 May 26;27:e66733. doi: 10.2196/66733 (PMC12149780; doi:10.2196/66733)
Supplement: Multimedia Appendix 2 [file jmir_v27i1e66733_app2.pdf]

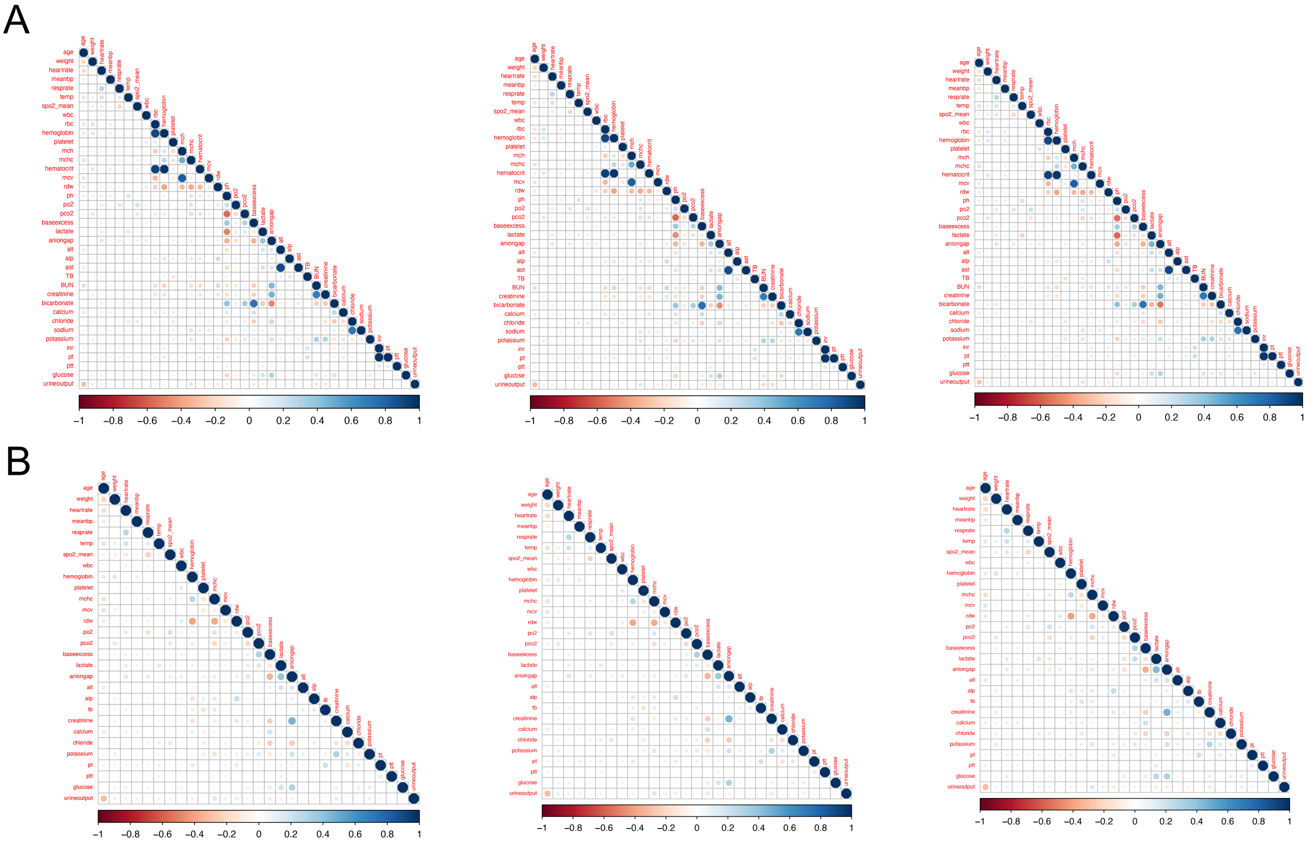

Figure S1. Correlation analysis of continuous variables in the MIMIC-IV training (A) and internal validation (B) sets.

This figure illustrates the correlation analysis of continuous variables from the MIMIC-IV database, separately for the training (A) and internal validation (B) sets. Variables with a Pearson correlation coefficient greater than 0.5 were excluded from further analysis to mitigate multicollinearity. The heatmaps display pairwise correlations between variables, with color and dot size representing the direction and magnitude of the correlation, respectively. Blue indicates positive correlations, while red indicates negative correlations.

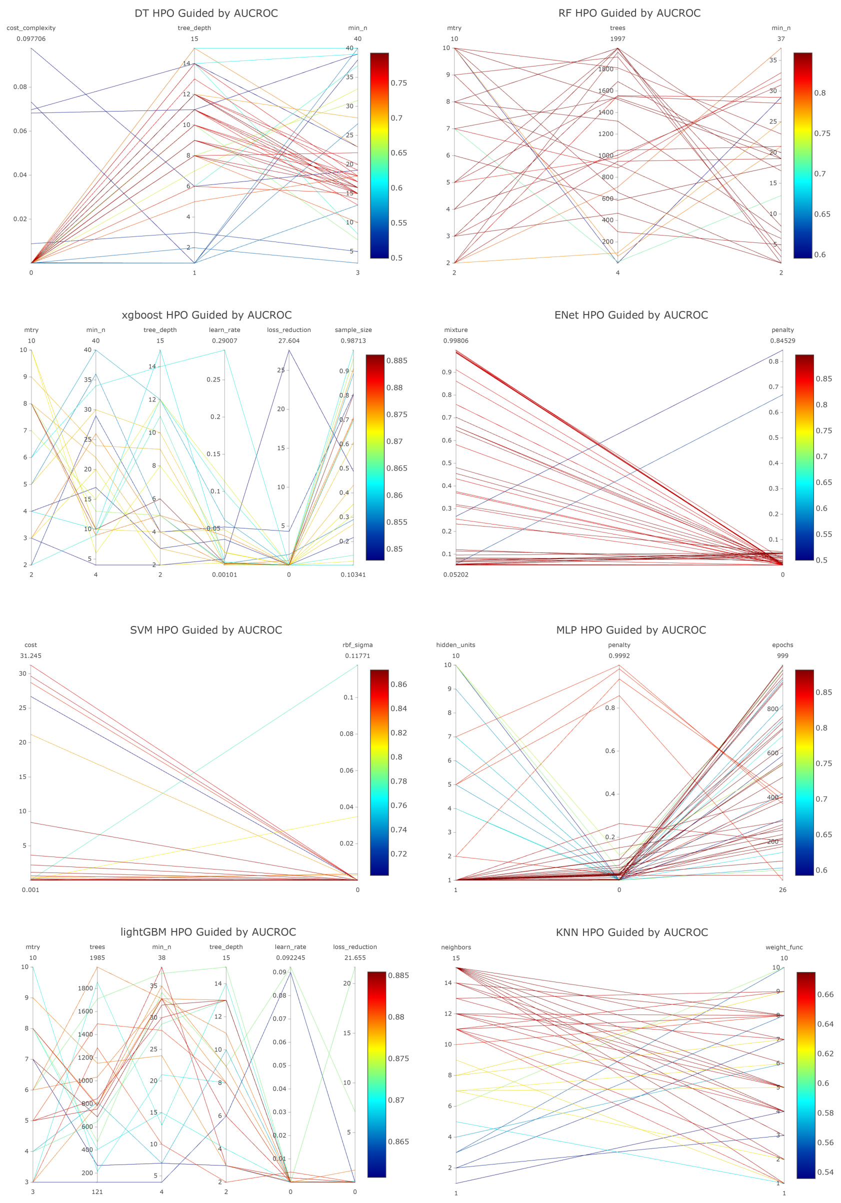

Figure S2. Hyperparameter optimization (HPO) guided by AUC-ROC for various machine learning models.

This figure presents the results of hyperparameter optimization (HPO) guided by the Area Under the Curve - Receiver Operating Characteristic (AUC-ROC) for eight machine learning models: Decision Tree (DT), Random Forest (RF), XGBoost, Elastic Net (ENet), Support Vector Machine (SVM), Multi-Layer Perceptron (MLP), LightGBM, and K-Nearest Neighbors (KNN). Each panel uses parallel coordinate plots to illustrate the effects of various hyperparameters on the AUC-ROC score. The color scale represents the AUC-ROC values, with red indicating higher AUC-ROC scores and blue indicating lower values.

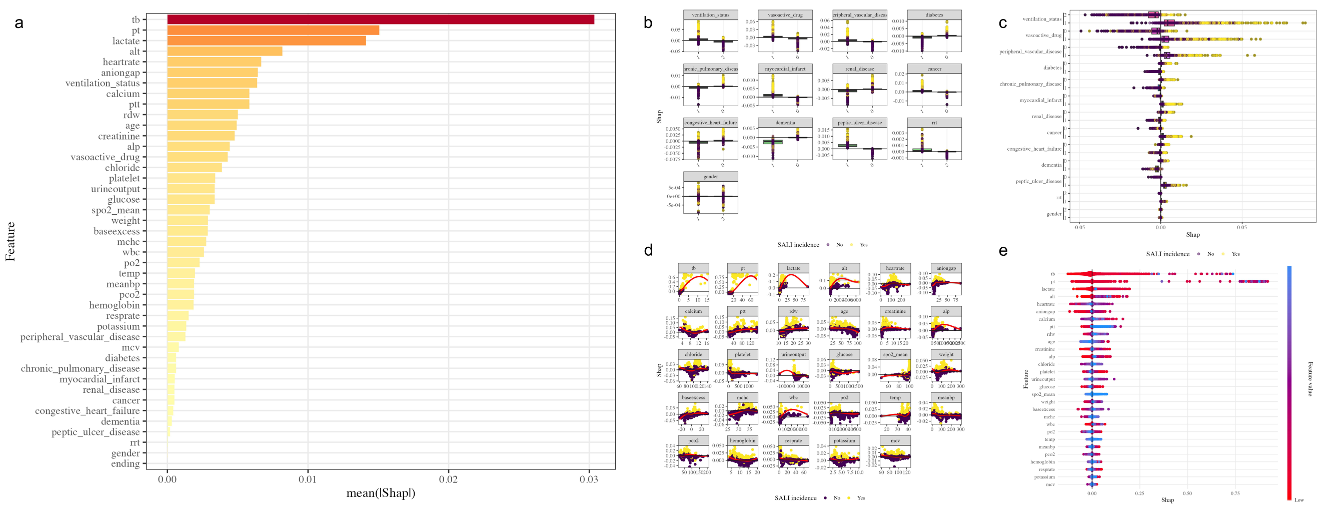

Figure S3. Feature importance and SHAP analysis of model predictions for LightGBM.

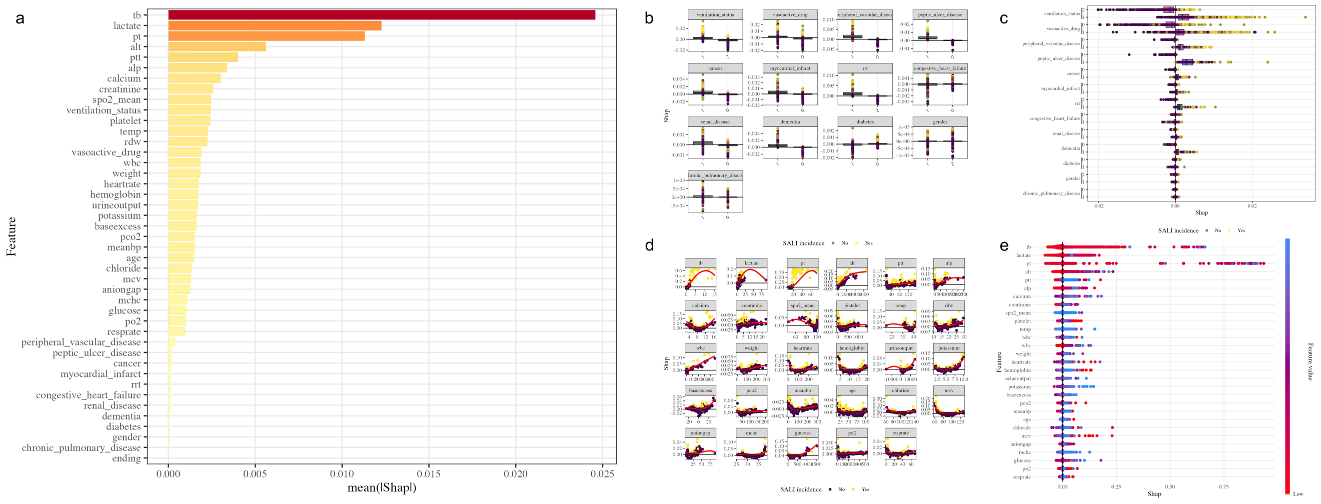

Figure S4. Feature importance and SHAP analysis of model predictions for RF.

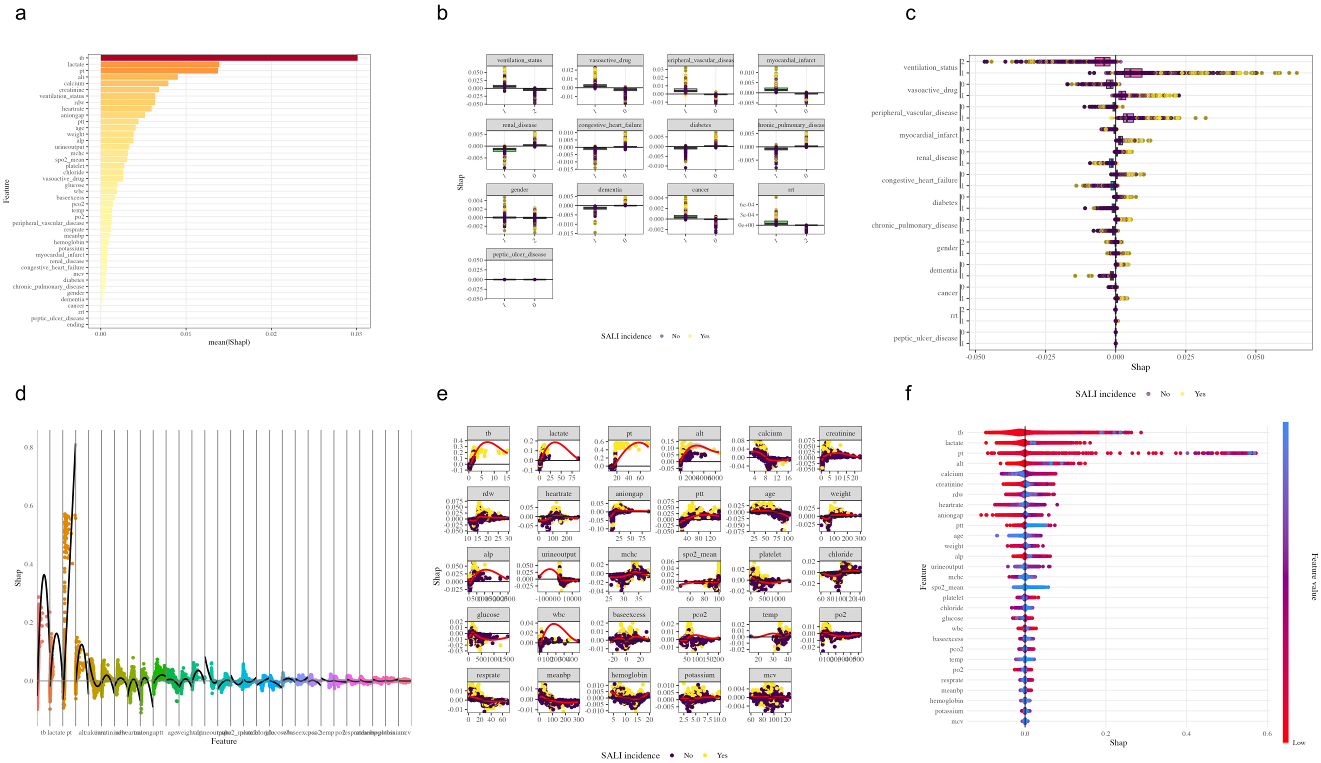

Fig. S5 Feature importance and SHAP analysis of model predictions for XGBoost. (a) Bar plot of mean absolute SHAP values, ranking features by their overall importance in the model's predictions. (b) SHAP summary plots for categorical variables, showing the distribution of SHAP values for key features across the dataset, highlighting the impact of each categorical feature on model output. (c) SHAP dependence plots for categorical variables, illustrating the relationship between these features and the model's prediction of SALI. (d) SHAP summary plots for continuous variables, displaying the distribution of SHAP values and capturing the impact of each continuous feature on model output. (e) SHAP dependence plots for continuous variables, demonstrating how individual continuous features influence the model's predictions, along with potential interaction effects with other features.

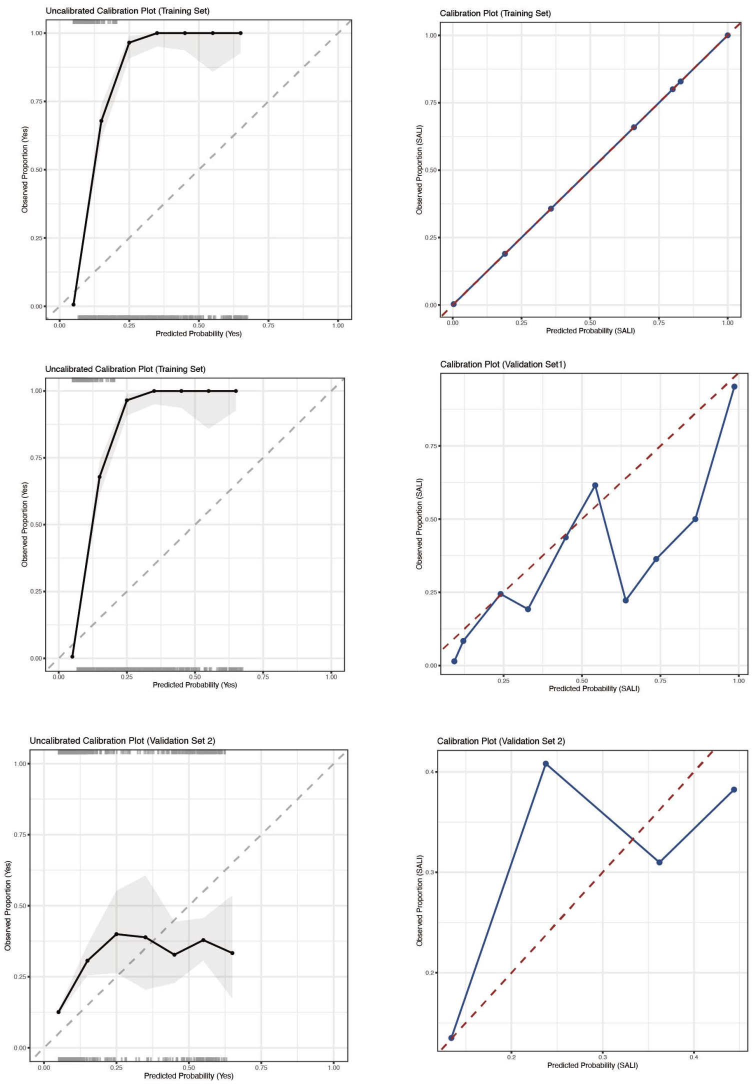

Figure S6. Calibration Plots Before and After Calibration for the Training and Validation Sets
