# Supplementary material for: Supervised Machine Learning Models for Predicting Sepsis-Associated Liver Injury in Patients With Sepsis: Development and Validation Study Based on a Multicenter Cohort Study
Source: J Med Internet Res. 2025 May 26;27:e66733. doi: 10.2196/66733 (PMC12149780; doi:10.2196/66733)
Supplement: Multimedia Appendix 3 [file jmir_v27i1e66733_app3.pdf]

Figure 2

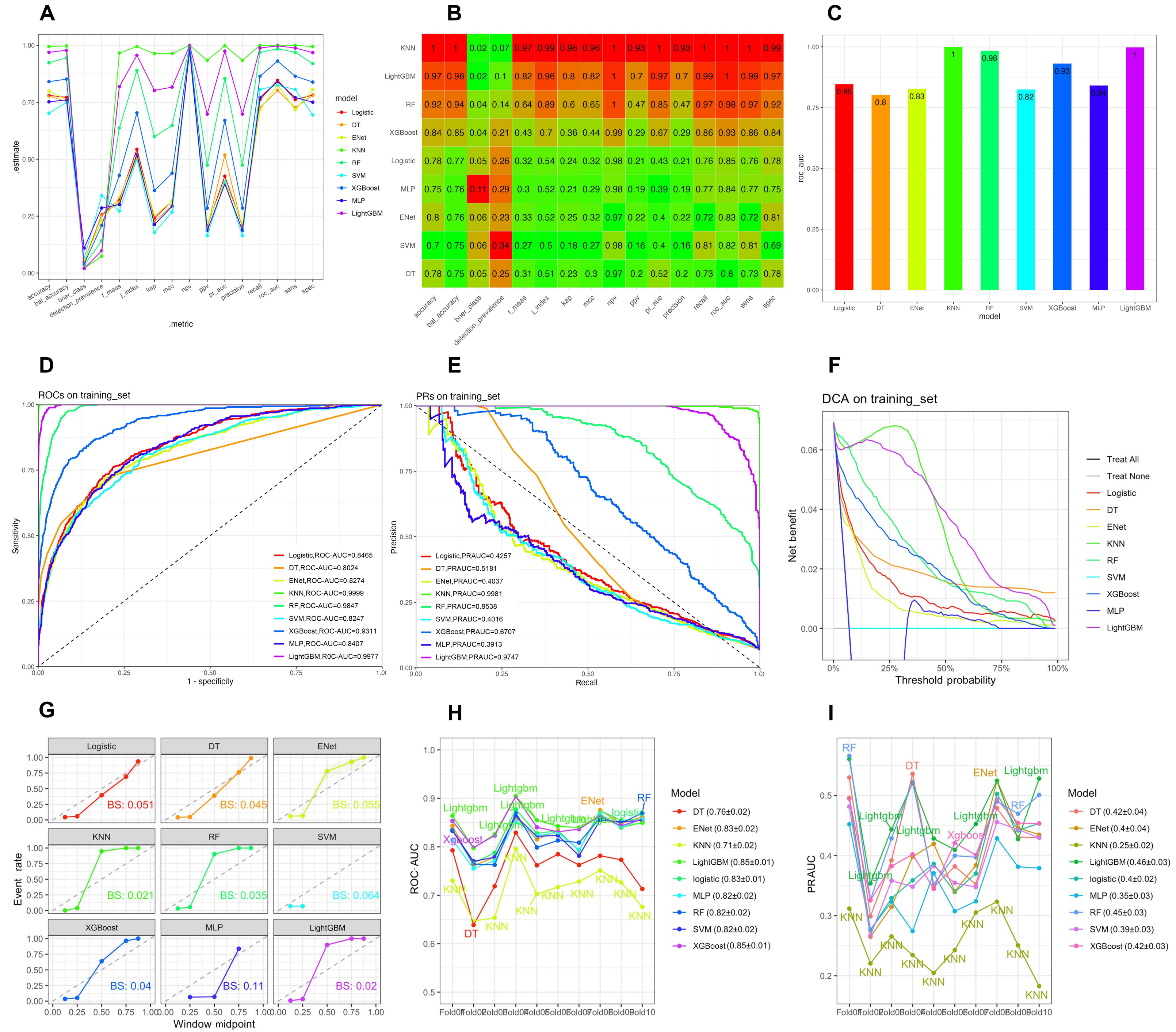
A
model
Logistic
DT
ENet
KNN
RF
SVM
XGBoost
MLP
LightGBM
.estimate
.metric
B
C
roc_auc
model
D
ROCs on training_set
Sensitivity
1 - specificity
Logistic,ROC-AUC=0.8465
DT,ROC-AUC=0.8024
ENet,ROC-AUC=0.8274
KNN,ROC-AUC=0.9999
RF,ROC-AUC=0.9847
SVM,ROC-AUC=0.8247
XGBoost,ROC-AUC=0.9311
MLP,ROC-AUC=0.8407
LightGBM,ROC-AUC=0.9977
E
PRs on training_set
Precision
Recall
Logistic,PRAUC=0.4257
DT,PRAUC=0.5181
ENet,PRAUC=0.4037
KNN,PRAUC=0.9981
RF,PRAUC=0.8538
SVM,PRAUC=0.4016
XGBoost,PRAUC=0.6707
MLP,PRAUC=0.3913
LightGBM,PRAUC=0.9747
F
DCA on training_set
Net benefit
Threshold probability
Treat All
Treat None
G
Event rate
Window midpoint
BS: 0.051
BS: 0.045
BS: 0.055
BS: 0.021
BS: 0.035
BS: 0.064
BS: 0.04
BS: 0.11
BS: 0.02
H
ROC-AUC
Model
DT (0.76±0.02)
ENet (0.83±0.02)
KNN (0.71±0.02)
LightGBM (0.85±0.01)
logistic (0.83±0.01)
MLP (0.82±0.02)
RF (0.82±0.02)
SVM (0.82±0.02)
XGBoost (0.85±0.01)
I
PRAUC
Model
DT (0.42±0.04)
ENet (0.4±0.04)
KNN (0.25±0.02)
LightGBM (0.46±0.03)
logistic (0.4±0.02)
MLP (0.35±0.03)
RF (0.45±0.03)
SVM (0.39±0.03)
XGBoost (0.42±0.03)

Figure 3

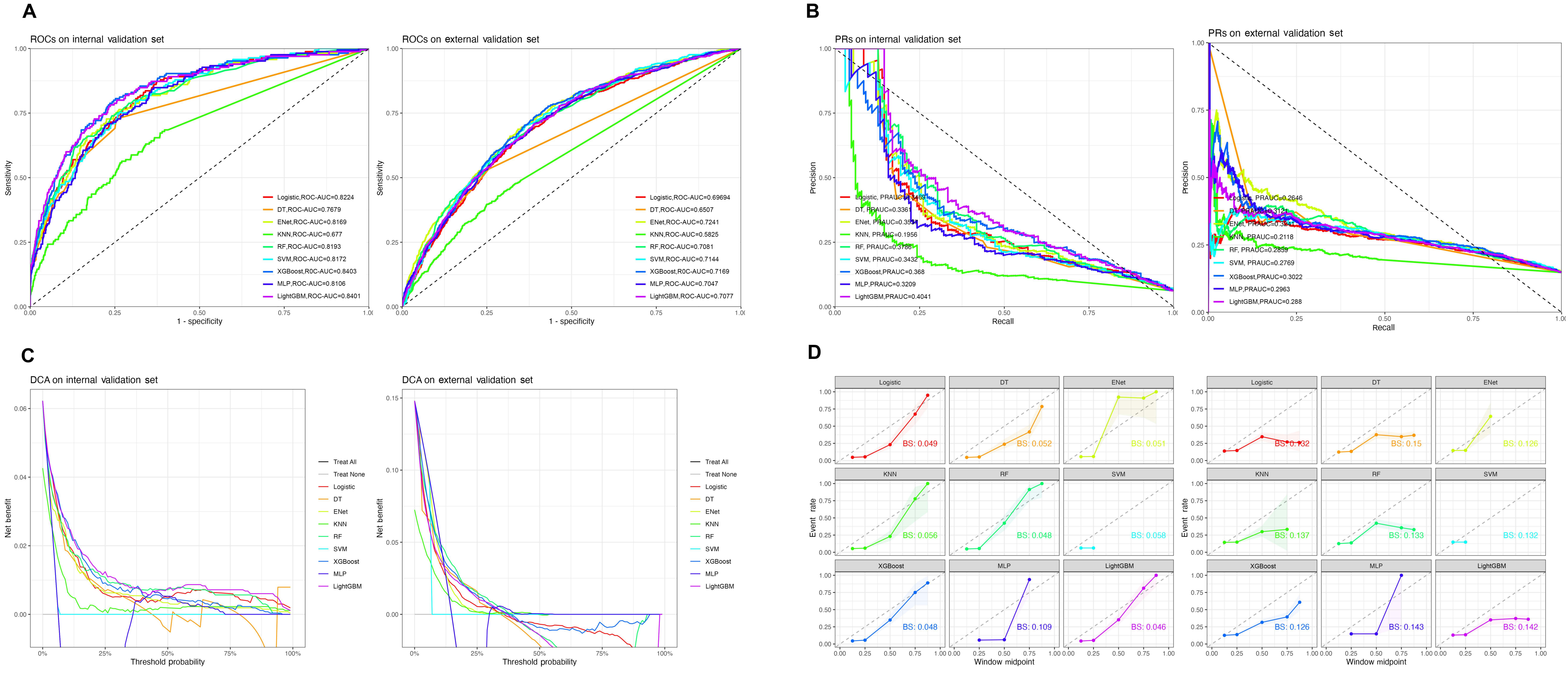
A
ROCs on internal validation set
Sensitivity
1 - specificity
Logistic,ROC-AUC=0.8224
DT,ROC-AUC=0.7679
ENet,ROC-AUC=0.8169
KNN,ROC-AUC=0.677
RF,ROC-AUC=0.8193
SVM,ROC-AUC=0.8172
XGBoost,ROC-AUC=0.8403
MLP,ROC-AUC=0.8106
LightGBM,ROC-AUC=0.8401
ROCs on external validation set
Logistic,ROC-AUC=0.69694
DT,ROC-AUC=0.6507
ENet,ROC-AUC=0.7241
KNN,ROC-AUC=0.5825
RF,ROC-AUC=0.7081
SVM,ROC-AUC=0.7144
XGBoost,ROC-AUC=0.7169
MLP,ROC-AUC=0.7047
LightGBM,ROC-AUC=0.7077
B
PRs on internal validation set
Precision
Recall
DT, PRAUC=0.3361
KNN, PRAUC=0.1956
SVM, PRAUC=0.3432
XGBoost,PRAUC=0.368
MLP,PRAUC=0.3209
LightGBM,PRAUC=0.4041
PRs on external validation set
Logistic, PRAUC=0.2646
KNN, PRAUC=0.2118
RF, PRAUC=0.2859
SVM, PRAUC=0.2769
XGBoost, PRAUC=0.3022
MLP,PRAUC=0.2963
LightGBM,PRAUC=0.288
C
DCA on internal validation set
Net benefit
Threshold probability
Treat All
Treat None
Logistic
DT
ENet
KNN
RF
SVM
XGBoost
MLP
LightGBM
DCA on external validation set
D
Logistic
DT
ENet
KNN
RF
SVM
XGBoost
MLP
LightGBM
Event rate
Window midpoint
BS: 0.049
BS: 0.052
BS: 0.051
BS: 0.056
BS: 0.048
BS: 0.058
BS: 0.048
BS: 0.109
BS: 0.046
BS: 0.15
BS: 0.126
BS: 0.137
BS: 0.133
BS: 0.132
BS: 0.126
BS: 0.143
BS: 0.142

Figure 4

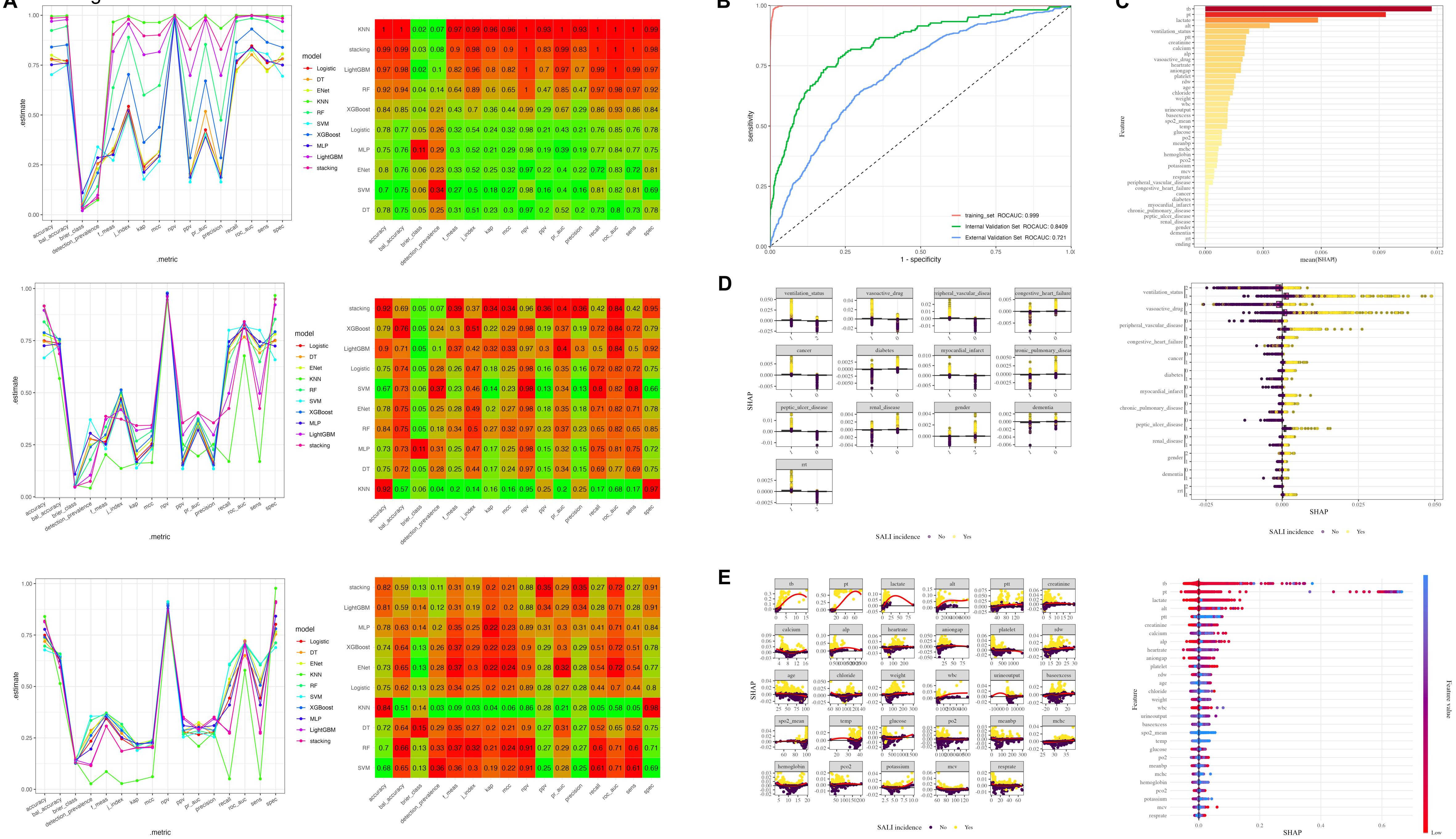
A
B
C
D
E
model
Logistic
DT
ENet
KNN
RF
SVM
XGBoost
MLP
LightGBM
stacking
.estimate
.metric
accuracy
bal_accuracy
brier_class
detection_prevalence
f_meas
j_index
kap
mcc
npv
ppv
pr_auc
precision
recall
roc_auc
sens
spec
sensitivity
1 - specificity
training_set ROCAUC: 0.999
Internal Validation Set ROCAUC: 0.8409
External Validation Set ROCAUC: 0.721
Feature
mean(|SHAP|)
SHAP
SALI incidence
No
Yes
Feature value
Low
